# Characteristics of Complex Systems in Sports Injury Rehabilitation: Examples and Implications for Practice

**DOI:** 10.1186/s40798-021-00405-8

**Published:** 2022-02-22

**Authors:** Kate K. Yung, Clare L. Ardern, Fabio R. Serpiello, Sam Robertson

**Affiliations:** 1 Institute for Health and Sport, Victoria University, Melbourne, Australia; 2 Musculoskeletal and Sports Injury Epidemiology Centre, Department of Health Promotion Science, Sophiahemmet University, Stockholm, Sweden; 3 Sport and Exercise Medicine Research Centre, La Trobe University, Melbourne, Australia; 4 Department of Family Practice, University of British Columbia, Vancouver, Canada

**Keywords:** Complexity, Return to sport, Return to play, Decision making, Machine learning, Bayesian network

## Abstract

Complex systems are open systems consisting of many components that can interact among themselves and the environment. New forms of behaviours and patterns often emerge as a result. There is a growing recognition that most sporting environments are complex adaptive systems. This acknowledgement extends to sports injury and is reflected in the individual responses of athletes to both injury and rehabilitation protocols. Consequently, practitioners involved in return to sport decision making (RTS) are encouraged to view return to sport decisions through the complex systems lens to improve decision-making in rehabilitation. It is important to clarify the characteristics of this theoretical framework and provide concrete examples to which practitioners can easily relate. This review builds on previous literature by providing an overview of the hallmark features of complex systems and their relevance to RTS research and daily practice. An example of how characteristics of complex systems are exhibited is provided through a case of anterior cruciate ligament injury rehabilitation. Alternative forms of scientific inquiry, such as the use of computational and simulation-based techniques, are also discussed—to move the complex systems approach from the theoretical to the practical level.

## Key Points

- Complex systems have distinct properties, such as nonlinearity, emergence and adaptation. Sixteen features of complex systems have been identified in sports injury rehabilitation.
- Rehabilitation practitioners may connect complex systems theory with their operations in the sports setting.

## Challenges in Return to Sport Decision Making

Return-to-sport (RTS) can challenge health professionals, coaches (i.e., practitioners) and athletes. In competitive sports, where marginal gains in performance are sought, athletes and practitioners often weigh risks and benefits when making the RTS decisions. In a team sports setting, full availability of players allows greater flexibility in tactical planning, such as deciding the best team formation based on the opponent’s playing style. Player availability is linked to performance [1–3] and could reduce the financial burden on the team [4, 5].

Research on RTS decision making largely focuses on identifying a criteria list based on biological factors and on whether the athlete has returned to baseline performance level (e.g., Grindem et al. [6], Stares et al. [7], and Kyritsis et al. [8]). This approach has assisted practitioners in being transparent in the decision process, for instance, to grant a medical clearance. However, underlying complexity and the high degree of interlinks, independencies, and temporal components also need consideration. For example, the same criteria may not apply to athletes of a different mental state, age group or playing level. Furthermore, non-linearity is commonly seen in the context of sports. As an example, most football fans would know that a team composed of the best-skilled players, does not necessarily produce the best performance. Instead, the outcome is highly dependent on the interplay of tactical, physiological, social and even emotional factors. Similarly, it may be beneficial to view RTS more than simply addressing a set of predefined RTS criteria, or achieving an arbitrary numerical change in a performance test.

To address these limitations and objectives, we propose an approach using the complex systems theory. Recent work from Bittencourt et al. [9] has raised awareness of the theory and more could be done to clarify the characteristics of complex systems and to increase the practical utility of the complex systems approach. Consequently, this paper builds on the work of Bittencourt et al. [9] and aims to (1) clarify the terminologies in the complex systems approach and adapt them for sports, (2) provide examples relevant to rehabilitation and (3) introduce tools that can model the complexity and increase practical utility in applied settings.

## What is a Complex Systems Approach?

### A Complex Systems Approach to Decision Making in Sports Medicine

The complex systems theory, with more than 50 years of history [10], acknowledges the multifaceted nature of sports and seeks to understand the interactions among different factors and the outcomes of the systems [9, 11]. Complex systems are dynamic, open systems [12]. They are characterised by non-linearity due to feedback loops and interaction among the factors. This means that outputs are not always proportional to the inputs, and a small adjustment may lead to a large change in the systems and vice versa [13].

**Fig. 1:**
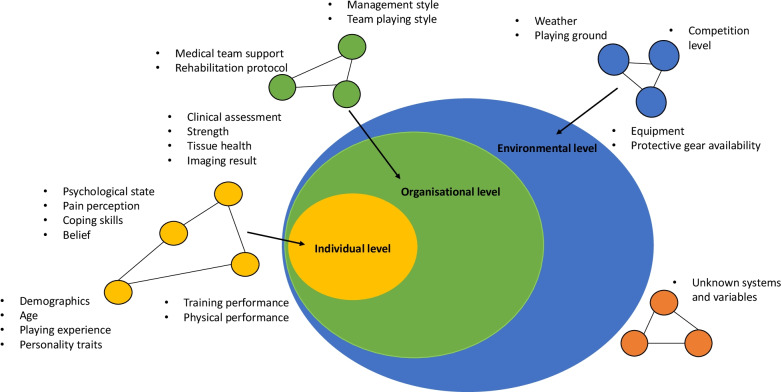
A multilevel system map with factors related to return to sport decision in anterior cruciate ligament injury

In complex systems, factors that interact with each other to form the systems are known as units [12]. In the context of RTS, these units could include age, wellness, biological healing of injured tissue, stress, external pressure and injury history. The units interact and define the space and dimension of the systems [14]. Consequently, different systems within systems emerge. These systems may be categorised based on their nature, for example, biomechanical, physiological and psychological. They may also be hierarchical and of multiple levels, namely individual, organisational and environmental (see Fig. 1). The individual level represents factors related to the individual athlete, from tissue healing to personal traits. The organisational level represents external factors related to the sporting club, organisation and support team, e.g., the coaching and medical team. The environmental level covers factors beyond the organisational level, such as the weather, playing schedule and competition level.

In recent years, the complex systems approach has gained momentum and has been used to understand sports injury occurrence [9, 15] and behaviour in sports performance [16–19]. However, the terminologies used in complex systems are often less familiar to practitioners and could be easily confused with merely *complicated* or *multifactorial.* Most studies recognize the importance of considering multiple factors in determining readiness for RTS or in the context of injury recognition [6, 8, 9, 20–26], but more work is required to raise awareness on why the lens of complex systems approach should be adopted by practitioners in rehabilitation.

### Applying a Complex Systems Model for ACL rehabilitation

This paper provides examples based on the 16 common features of complex systems recently illustrated by Boehnert et al. [27]. They are adapted for the context of sports in Table 1, with examples illustrated mainly from an anterior cruciate ligament (ACL) injury.

An ACL injury is used here as the case illustration as it is a serious injury that may threaten the career of an athlete [28, 29]. The estimated annual medical cost associated with ACL reconstruction surgery in Australia was over A$75 million per year [30]. Currently, there is no consensus regarding the optimal functional rehabilitation criteria [20] and objective physiological RTS criteria [31]. Despite ACL injuries being one of the most researched topics in the sports medicine literature [32], the re-injury risk of ACL remains high [33, 34]. The complexity within ACL RTS may be explained at the individual, organisational and environmental levels.

**Table 1:** The 16 common features of complex systems adapted for return-for-sport

Characteristics	Definition	Example
1. Feedback	Units in a complex system are mutually interacting and output is fed back and becomes a new input [70]. The feedback could be positive or negative. For example, positive feedback increases the rate of change while negative feedback works by reversing the direction of change.	Rehabilitation training leads to tissue adaptations, which improves physical fitness and performance (positive feedback). However, maladaptation can occur (e.g., alteration in neuromuscular control and muscle damage), leading to suboptimal response which may delay progress (e.g., delayed onset of muscle soreness). This acts as negative feedback for the systems, signalling the training intensity was too high.
2. Emergence	Emergent properties arise from the interactions of its units. The units serve as the building blocks for patterns to arise at higher levels [71].	After an ACL injury, injured athletes often train separately from the squad and have a different training regime. During this time of relative isolation and hardship, the athlete may build up a high level of resilience.
3. Self-organisation	Systems may order themselves spontaneously to form patterns and achieve an optimal or stable state [14].	ACL is a key sensorimotor system for postural control, which helps to maintain and control upright posture [72]. Following an ACL injury, the brain activation profile will be affected and shifts toward a visual-motor strategy, as opposed to a sensory-motor strategy. Instead of relying on movement and spatial awareness, people with ACL deficiency may rely more on the visual system, especially under challenging dynamic task constraints [73]. This is an example of how the sensorimotor system self-organises to compensate for the loss of ACL.
4. Levers and hubs	Levers and hubs are key structures in the systems that play a crucial role in how the systems will behave. Identifying them could allow interventions in the systems effectively [27].	There are exceptional factors that are influential in the RTS process and altering them may lead to rapid gain. In ACL rehabilitation, intense rehabilitation and patient motivation are established levers and hubs that may underpin a positive outcome following ACL rehabilitation [74].
5. Non-linearity	Outputs are not always proportional to the inputs. Small changes may lead to a large change in the systems and vice versa [14].	The same training stimulus can create a large recovery response (e.g., delayed onset of muscle soreness) on the first training session, but not subsequent training. This is because the body can non-linearly adjust to the training stimulus after the first session. The response exhibits a non-linear behaviour where the outcome (i.e., training response) is not proportional to the input (i.e., training stimulus).
6. Domains of stability	Many systems are dynamic however may eventually converge to a stable state. This stability will be maintained unless there is a significant perturbation [70].	Balance and proprioceptive training are often included in the ACL rehabilitation protocol. However, balance and technique training may not be effective in changing an athlete’s knee joint kinematics or decreasing external knee moments during pre-planned and unplanned side-stepping [75]. Similarly, gait mechanics are also difficult to modify even after completion of rehabilitation training and restoration of muscle strength [76]. This may be because the systems have achieved a domain of stability and the parts of the systems are well-entrenched, making it very difficult or near impossible to change. Once the systems have achieved a state of stability, they could only be altered when the stimulus is strong enough to push them through the tipping point [70].
7. Adaptation	Components or actors within the systems are capable of learning and evolving in response to the changes in the environment [70].	Some people with ACL deficiency may exhibit increased knee flexion at early stance and reduced extension in mid to late stance [77]. This is an adaptation that allows hamstrings to be efficient synergists to the ACL in walking [78, 79] and to reduce the anterior translation force of the tibia [77]. This represents how the body adapts to ACL deficiency by bringing changes within the systems. The adaptation appears to happen autonomously, unconsciously, and without explicit programming.
8. Path dependency	Events and actions that occurred previously influence future states and decisions [27].	ACL rehabilitation usually follows a path and one can only progress to the next stage by meeting a set of criteria. For example, in the early rehabilitation phase, progressive weight-bearing allows the knee joints to acclimatise to increased load and assist in the development of a normal gait pattern [80, 81]. Plyometric training is only incorporated if full range of motion (ROM), sufficient strength base, and flexibility are demonstrated. For on-pitch rehabilitation, activities should begin with simple drills and advance to more complex exercises [80]. A control-chaos continuum (CCC) could be followed on-field, where rehabilitation training constraints progress from high control to high chaos [82].
9. Tipping point	If the perturbation of a system goes beyond a certain threshold, there will be a phase transition in the system's behaviour which may not be reversible [70].	In ACL rehabilitation, one of the early goals is to strengthen lower limb muscles to minimise muscle atrophy [83]. Squat exercise may be used as a training stimulus (perturbation) and it may cause micro-tears and inflammation of the muscle fibres (system deviates from the stable state). The neuromuscular system will repair and adapt (system returns to a stable state), leading to muscle hypertrophy [84]. However, if the intensity and volume exceed the capacity of the soft tissue, there will be a loss in stability (e.g., quadriceps muscle strain) and an inability to relax back to the previous stable state automatically. There will be a change in system behaviour (i.e., re-injury [85]).
10. Change over time	Systems are dynamic and can evolve over time. This is because they constantly interact and negotiate with the environment, leading to continuous change [70].	Psychological characteristics of athletes can change during the ACL rehabilitation process and affect how they cope with RTS and future injury [86]. In the physical performance aspect, training capacity evolves and generally declines with age [87]. For example, the heart rate maximum during exercise declines with age [88]. Maximal oxygen consumption is inversely and strongly related to age for active and endurance-trained populations [89].
11. Open system	Complex systems are considered open as it is difficult to define their boundary. The systems interact with the environment and are also being influenced by the environment continuously. In contrast, closed systems are systems where the influence of the environment on them is negligible [14].	The size of the systems could hardly be defined, as things in the environment that are seemingly small may also influence them. For example, a wet training ground affects the ground reaction force and movement strategy for athletes during running [90]. Shoe designs and types of playing surfaces are related to ACL injury risk due to the shoe-surface friction [91]. Playing music during rehabilitation training may reduce the perception of physical effort during training and improve physical performance by delaying fatigue or increasing work capacity [92, 93].
12. Unpredictability	Due to non-linearity and emergence properties, it is difficult to predict how the systems will evolve [9].	Precise forecasting of when an athlete should RTS is challenging. It is difficult to predict the estimated time for recovery as there is unpredictability on how the systems evolve. For example, how will the motivation of the athlete change throughout rehabilitation? How will the change in a personal relationship affect the performance of the athlete? In some cases, it is impossible to gather, store, and use all of the information about the state of complex systems at one point to predict the outcome.
13. Unknowns	There are always units that influence the systems which are either unknown or could not be observed or measured. Therefore, it may seem that the systems evolved unpredictably [9].	There are factors that decisions makers may not be aware of during the ACL rehabilitation due to different reasons, for example, limited knowledge (e.g., how a genetic variant is associated with ACL rehabilitation and injury risk?), technology constraints (e.g., how reliable are the measurement tools?), insufficient resources (e.g., is it possible to measure everything?), bias and issues that stakeholders have been unaware of.
14. Distributed control	Control of a system is distributed across different parties and no one has complete control over the systems [9]. There is no top-down control approach as the process is not controlled by a single factor at a superior level.	The success of ACL rehabilitation is determined by all interacting units, from biological graft healing at the microscopic level, to intra-personal factors (clinical assessment, functional test, and biopsychosocial factors), and inter-personal factors at the macroscopic level. No single factor in isolation could determine the success of the outcome.
15. Nested system	There are nested hierarchies within the complex systems, forming systems within systems [27].	ACL rehabilitation itself exhibits nest hierarchies in the following order: Cell > muscle > brain > inter-personal > family and friends > organization > environment. At the cell level, shortly after graft implantation, fibrous scar tissue will be formed between the graft and host bone [94], followed by ligamentization [95]. At the muscular system level, quadriceps muscle atrophy and dysfunction are commonly observed after ACL reconstruction and are often associated with altered movement pattern [96, 97], possibly due to alterations at the brain (motor cortex) level and neurophysiological changes in muscles [98–101]. At the intrapersonal level, physiological cardiac adaptation [102] and aerobic fitness [103] are all substantially reduced after an ACL injury. At the interpersonal level, social support plays a key role in regaining confidence and eradicating fear of re-injury throughout rehabilitation [104–106].
16. Multiple scales and levels	Multiple perspectives are required when viewing complex systems. The systems are three dimensional and interactions within the systems often occur at different scales and levels [27].	Rehabilitation can be considered on the biological level, psychosocial level or performance level. There is more than one domain involved and often the systems have to be understood from multiple perspectives.

## Implications for Practice and Future Research

By illustrating the features of complex systems with a common sports injury, we highlight their practical utility in RTS. The complex systems approach provides a theoretical framework for interpreting the patterns that emerge from biopsychosocial and other external factors. In ACL rehabilitation, conducting independent clinical tests and functional assessments may provide useful information regarding the athletes’ physical and mental status. However, a complex systems approach facilitates a more complete picture of the problem and an increased awareness of how different factors may interact.

There are two challenges on using the complex systems approach: (1) the high degree of complexity may deter practitioners who do not have formal training in handling large and complex datasets from using this approach, (2) Unlike studying in a controlled laboratory environment, it is near impossible to isolate a portion of the larger systems (i.e., isolation of the biological healing process from broader biopsychosocial factors). Fortunately, many computer-based decision support systems now have the capability of incorporating features of complex systems in their design and utility. For example, to operationalise one of the above features, “change over time”, the working model can allow flexibility in updating the baseline and encourage repeated testing at multiple time points during the rehabilitation. We believe practitioners who develop an understanding of complex systems will be well-positioned to efficiently articulate their needs with analysts and ultimately develop decision support systems that inform best practices (e.g., RTS decision making).

Computer simulation (e.g., agent-based modelling), machine learning and Bayesian network (BN) analyses are all potential tools for analysing both non-complex or complex systems [35]. These methods can consider the dynamic interaction at multiple levels simultaneously, consequently viewing RTS more completely and supporting decision making. These analytical tools may help to achieve the following: (1) allow practitioners to study and compare the potential outcome (e.g., likelihood of reinjury) of different decisions that are otherwise almost impossible to test safely in real life, (2) increase the decision efficiency by learning from previous experience and streamlining data from multiple sources and formats, (3) identify patterns in data that may cause a certain outcome.

These techniques can be used to construct clinical decision support systems, which may complement or be superior to human decisions. In a review of seventy studies, a decision support system improved clinical practice in 68% of trials [36]. These decision support systems have also provided more accurate diagnoses than human experts in some medical fields [37, 38]. Yet, the application of these approaches in RTS is still scarce in the literature. As such, we have provided a vignette here to outline how machine learning techniques and Bayesian networks could be applied to support RTS decision making: a 30-year-old professional female football player tore her hamstring 10 days ago during the season and a grade II hamstring strain was diagnosed. There is an important match in 2 weeks and there are six relevant questions, as covered in the below sections, which the practitioners and the coach would like to ask. Ultimately, the coach would like to know as early as possible about the availability of the player so that they could plan the players’ list and hence the game strategy.

### Machine Learning Techniques

As a subfield of artificial intelligence (AI), machine learning focuses on the use of data to train algorithms that can make classifications or predictions [39, 40]. That is, it could recognise new meaningful correlations, patterns and trends in a large amount of data [41]. Not only are machine learning techniques suitable for non-complex analysis, but they can also accommodate multi-dimensional analysis in sport [42, 43]. New data could also be input into the model for it to learn and improve the task, leading to refinement of skills [40].

The goals of machine learning techniques in sports medicine setting can be divided into predictive and descriptive modelling [44]. Specifically, predictive modelling can be used for injury prognosis, diagnosis, and rehabilitation planning. Descriptive modelling can be used to characterize the general property of an injury, such as its severity, as well as include hypotheses of causality. However, as with traditional statistical approaches, machine learning techniques are simply a method for analysing the data, providing a prescriptive or descriptive output. For understanding and estimating causal relationships, appropriate study designs are required, for example, randomised controlled trials. Machine learning is often characterised by five major approaches (i.e., association, classification, clustering, relationship modelling and reinforcement learning), each having already been applied for injury risk assessment and/or performance prediction in sports [45–49]. Each of these approaches could serve as the methods to answer questions relevant to RTS.

#### Question 1: Should the Athlete Progress to Full Training?

*Scenario* The athlete has completed 10 days of rehabilitation training. The practitioners would like to assess whether the athlete is ready to progress to full training. An association approach could be used here, using the rule-based system (Table 2).

**Table 2:**
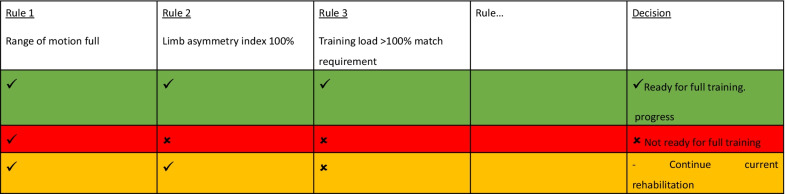
The association approach to determine should the athlete progress to full training

Rule-based approaches identify meaningful and frequent patterns between variables in a large dataset [50]. Often less identifiable by the practitioner, the rules may help them identify patterns that indicate optimal rehabilitation combinations of variables by flagging both commonly occurring and meaningful patterns in data.

In the above hypothetical example, a multivariate analysis of rules associated with a rehabilitation outcome is conducted. The model was set to only produce 3 categories of rules that contained the rehabilitation outcome as a result (i.e., ready for full training, not yet ready and unchanged). These could be the three rules most strongly associated with the rehabilitation outcome. A tick represents the presence of the context within the rule. The system could identify the number of rules required based on previous rehabilitation experience and to implement the rules when the complexity of the content is beyond human brain capacity. An increased number of rules may better represent complexity; however, it may potentially make the solution more difficult to operationalize practically.

#### Question 2: What is the Likelihood that the Athlete Could Return to the Pre-injury Level Given the Current Level of Training?

*Scenario *There are only 2 weeks until an important match. The coach would like to know the likelihood that the athlete could return to pre-injury level by then. Given the volume of high-speed running training that the athlete has completed, a classification method could be used to identify the likelihood (Table 3).

**Table 3:** The classification approach to identify the likelihood for an athlete to RTS

Approach	Classification
Task	Supervised
Technique	Decision tree and random forest
Output type	Categorical or continuous Examples: ready to compete, not yet ready to compete
Application example	

A decision tree uses dichotomous divisions to create the classification algorithm. Representing the rules, the decision tree could be used to develop a clinical decision algorithm for RTS [49, 51]. Each node denotes a test on an attribute value and each branch represents an outcome of the test, with the leaves representing the class.

The above is a graphical representation of the decision tree that used a classification algorithm to identify the probability of RTS from a hamstring injury. Each node is associated with a rule condition, which branches off to the child node. In this example, the outcome of RTS is likely a non-linear relationship with the training volume and mental readiness, which is a characteristic of the complex systems approach (see Table 1, example 5). Using the classification approach may help to include non-linearity into analyses.

#### Question 3: When is the Athlete Expected to Return to Sport?

*Scenario* The coach would like to know when the athlete is expected to RTS based on the experience of the clinician and also accounting for the athlete’s age. Clustering technique could be used to analyse the past data.

Clustering allocates data points into groups that share similar or dissimilar features [52]. In RTS, this may be useful in the allocation of multiple athletes to training groups. This could be done for clinical presentation, playing position, demographics, or inter-and intra-personal factors.

Table 4 visualizes one of the multiple approaches to which injured athletes could be clustered. Each dot represents an injured athlete and is coloured based on their severity. Size represents a measure of each athlete’s age, with a larger size representing older age. They are further grouped into three different clusters, representing the severity and time to RTS. In this hypothetical example, the model output is the predicted days to RTS. However, it could also be designed to produce categorical outputs such as being ready to train or not yet ready to train.

**Table 4:** The clustering approach to identify when the athlete may return to sport

Approach	Clustering
Task	Unsupervised
Technique	K-nearest neighbours
Output type	Categorical Examples: RTS grade, days to RTS
Application example	

#### Question 4: The Athlete has a High Level of Mental Readiness. Would that Change the Level of Confidence About the Athlete’s Readiness to Play in an Important Game?

*Scenario* From the clustering approach, the coach has considered that the athlete may require at least 2 weeks to return to competition at pre-injury level. However, the coach noticed that the athlete had a high level of mental readiness, as reflected by relevant measures (e.g., Injury-Psychological Readiness to Return to Sport scale [53]). The coach would like to know how this new information, combined with the previous knowledge, may change the practitioner’s judgement. A relalationship modelling approach described below is used.

Relationship modelling involves estimating relationships between a dependent variable and one or more independent variables. Regression analysis, commonly used in the analysis, is also a type of relationship modelling technique and could be used with the complex systems approach. For example, it could be used for modelling the relationship between outcomes, such as match results [54] and injury incidence [45].

Table 5 shows a hypothetical example of how the confidence to RTS (y-axis) may be associated with the volume of high-speed running done (x-axis) and the mental-readiness score (size of the bubble). The level of mental readiness is denoted by the size of the bubble. A higher level of mental readiness is indicated with a larger size bubble and is in green colour. A lower level is indicated with a smaller size and is in red. The association could be multi-dimensional and could be constructed based on the number of inputs available, e.g., running speed, load accumulation, psychological readiness.

**Table 5:** The relationship modelling approach to identify the effect of mental readiness

Approach	Relationship modelling
Task	Supervised
Technique	Regression and neural networks
Output type	Continuous
Application example	

#### Question 5: What is the Optimal Sequence of Rehabilitation in a Case of Hamstring Injury Rehabilitation?

*Scenario* After reviewing the dataset, the coach and the clinician would like to explore how to further leverage the available data and identify adaptive personalized treatment plans in the future. Reinforcement learning may help to optimize the sequence of decisions that favour a long-term outcome. Reinforcement learning is described below.

Unlike supervised or unsupervised learning, reinforcement learning trains itself through trial and error to explore behaviours in the system that could maximize the reward [55]. This feature makes it suitable for solving sequential decision problems. In this clinical vignette, reinforcement learning could help to identify a personalized rehabilitation pathway for maximizing the reward (i.e., managing the injury or reaching the rehabilitation goal).

In the context of a hamstring injury (see Table 6), a practitioner has to decide when to initiate and adjust rehabilitation training, such as jogging, eccentric hamstring exercise, and high-speed running. Each decision affects the athlete’s rehabilitation outcome at the end of the program and the total days of absence. The rewards require practitioners’ input, such as comparing the intensity and volume of high-speed running to the pre-injury. The reliability of the treatment-quality estimate depends heavily on the amount of data that were used to train the algorithm used in the reinforced learning, and the extent to which the proposed and observed treatment policies agree.

**Table 6:** Use of reinforcement learning to optimise the sequence of rehabilitation

Approach	Reinforcement learning
Task	Not applicable
Technique	Markov decision process
Output type	No output variable
Application example	

### Bayesian Network

Besides the machine learning approach, Bayesian methods are becoming increasingly popular in the study of sports [56] and may contribute to RTS. Various forms of BN have been applied across different sectors, including medical [57–61], ecology [62–64] and transportation [65].

BN uses Bayesian inference for probability computations and can be visually presented using directed acyclic graphs. Arrows on the BN, known as directed arcs, indicate the direction of the influence [66]. These show how various discrete or continuous factors in RTS influence one another and the outcome in a graphical presentation [66]. BN allows calculation of the conditional probabilities of the outcome of a decision when the value of some of the factors has been observed. As new evidence is revealed, changes are brought to the conditional probability of the decision outcome [67].

#### Question 6: How Would the Sex of the Athlete Affect the Perceived ACL Injury Risk?

**Fig. 2:**
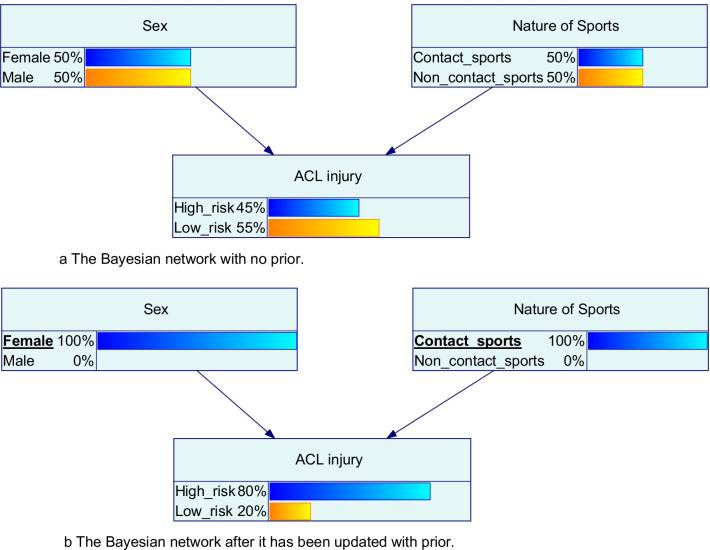
Illustration of a Bayesian network before (**a**) and after it has been updated with a prior (sex or/and nature of sport) (**b**). The outcome of the prediction (ACL injury risk) has changed as a result

*Scenario* The athlete has now recovered from the hamstring injury but is worried about the potential ACL injury risk. The coach wants to know how the sex of the athlete (prior) [as female] would affect how one perceives the ACL injury risk (outcome) [higher risk of ACL injury] (Fig. 2) [68], and how it may inform the potential consequence of a RTS decision.

**Fig. 3:**
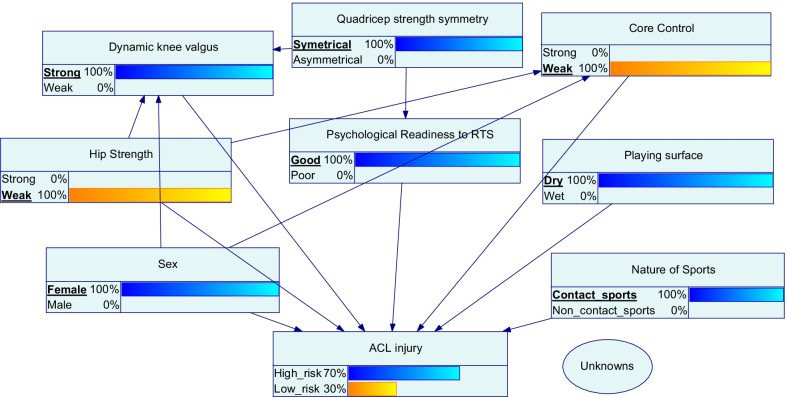
A hypothetical example of a Bayesian network with multiple priors for ACL injury risk

Only one prior is used here to explain the application for easier understanding. However, a BN can account for multiple variables to increase the accuracy of the model and to acknowledge the complex systems approach, as seen from a hypothetical example here in Fig. 3.

A BN could be operated in both directions, performing both predictive and diagnostic inference. As an example, a BN may provide the following information to support RTS decisions: (1) given the observation of the athlete’s rehabilitation markers, what is the likelihood for the athlete to perform at pre-injury level upon RTS? (2) to increase the likelihood to achieve certain outcomes of RTS, what is the combination of test results and/or observations required?

Logically, BN seems to fit into the requirement of RTS decisions, as often multiple unknown factors are involved in the process (e.g., how wellness may be associated with the injury risk). Although these unknown parameters are uncertain, they could be described by a probability distribution table, with information supplied by a domain expert or relevant literature.

Establishing a BN requires data and could be complemented by expert knowledge [66]. Expert knowledge allows the model to specify the decision options available and the utilities that the user is after. For example, decision-makers may decide if the utility (degree of satisfaction) of the RTS outcome is based on either maximising the team performance, minimising the risk of subsequent injury, or equilibrium between the two. However, this also implies that the quality of the model output would rely on the quality of the existing evidence and expert’s knowledge, which may be flawed or biased.

## Future Research

A shift towards a complex systems approach may help to view RTS more realistically. Future research should be mindful of the following issues:

1. The complex systems approach and the machine learning techniques cannot necessarily elucidate the causal mechanism. Based on Table 1, the characteristics of complex systems do not permit cause and effect relationships to be determined. However, that does not imply they are inappropriate for understanding a problem nor they are of low practical utility.
2. The accuracy of the computation relies heavily on the quality of the dataset and previous knowledge. For example, what is the association between different variables (e.g., age, playing style, previous injury history, culture, and lifestyle)? What is the potential effect of external factors (e.g., stress, financial pressure, lack of social support) on RTS progress and decision making? Currently, there is insufficient evidence on these aspects. High quality randomized controlled trials and longitudinal research that acknowledges the complex systems approach are required to observe regularities that are antecedent to the success of a rehabilitation program.
3. The RTS systems that researchers could construct would consist of what is available and known, rather than what is important. Some factors may be difficult to measure due to the availability of time, resources and their non-deterministic or qualitative nature [69]. For example, motivation for RTS during rehabilitation is important but often not measured due to difficulty obtaining accurate feedback. However, this is inevitable, as unknowns and unpredictability are characteristics of complex systems. Nevertheless, if possible, real data should be applied to prove the concept and provide useful output for practitioners, as the ultimate goal of embracing complex systems approaches in RTS is to produce findings closer to the real world.

## Conclusion

The complex systems approach has been applied to understand different aspects of sports science and medicine. This review has highlighted the characteristics and terminologies of complex systems, as exhibited by a case of ACL rehabilitation. When assessing the test result for clinical and functional tests, practitioners should also be aware of the dynamic systems evolving around the injury rehabilitation (refer to the examples in Table 1) and endeavour to understand the full picture. Future research may make use of computational modelling and machine learning techniques to identify the regularities of the pattern that emerges as a whole. A paradigm shift that results in the application of complex systems approach to understanding the RTS process and decision making should be encouraged.

## Data Availability

Not applicable.

## Abbreviations

ACL: Anterior cruciate ligament
AI: Artificial intelligence
BDN: Bayesian decision network
BN: Bayesian network
RTS: Return-to-sport
